# Cytogenetically Unrelated Clones in Acute Myeloid Leukemia Showing Different Responses to Chemotherapy

**DOI:** 10.1155/2016/2373902

**Published:** 2016-03-10

**Authors:** Kohei Kasahara, Masahiro Onozawa, Naohiro Miyashita, Emi Yokohata, Miho Yoshida, Minoru Kanaya, Mizuha Kosugi-Kanaya, Ryo Takemura, Shojiro Takahashi, Junichi Sugita, Akio Shigematsu, Mutsumi Takahata, Shinichi Fujisawa, Daigo Hashimoto, Katsuya Fujimoto, Tomoyuki Endo, Takeshi Kondo, Takanori Teshima

**Affiliations:** Department of Hematology, Hokkaido University Hospital, Sapporo 060-8638, Japan

## Abstract

We report a case of acute myeloid leukemia (AML) with two cytogenetically unrelated clones. The patient was a 45-year-old male who was diagnosed with acute monoblastic leukemia (AMoL). Initial G-band analysis showed 51,XY,+6,+8,inv(9)(p12q13)c,+11,+13,+19[12]/52,idem,+Y[8], but G-band analysis after induction therapy showed 45,XY,-7,inv(9)(p12q13)c[19]/46,XY,inv(9)(p12q13)c[1]. Retrospective FISH analysis revealed a cryptic monosomy 7 clone in the initial AML sample. The clone with multiple trisomies was eliminated after induction therapy and never recurred, but a clone with monosomy 7 was still detected in myelodysplastic marrow with a normal blast percentage. Both clones were successfully eliminated after related peripheral blood stem cell transplantation, but the patient died of relapsed AML with monosomy 7. We concluded that one clone was de novo AMoL with chromosome 6, 8, 11, 13, and 19 trisomy and that the other was acute myeloid leukemia with myelodysplasia-related changes(AML-MRC) with chromosome 7 monosomy showing different responses to chemotherapy. Simultaneous onset of cytogenetically unrelated hematological malignancies that each have a different disease status is a rare phenomenon but is important to diagnose for a correct understanding of the disease status and for establishing an appropriate treatment strategy.

## 1. Introduction

Cytogenetic alterations are considered to be useful markers to identify clones and to follow residual disease after treatment. Most hematologic malignancies have generally been believed to be monoclonal at initial presentation. Cytogenetic analysis usually shows one abnormal clone at initial presentation. Cytogenetically “related” clones, which have a chromosomal alteration in addition to the initial abnormality, sometimes coexist in one sample or appear during the clinical course and show a clonal evolution. In rare cases, clones with “unrelated” karyotypes were detected at diagnosis or at different times in the course of disease. Cytogenetically unrelated clones are uncommon findings in hematological disorders, occurring with frequencies of 4.3–6.5% in myelodysplastic syndrome (MDS), 1.1–3.7% in acute myeloid leukemia (AML), 0–0.6% in acute lymphoblastic leukemia (ALL), and about 7.3% in chronic lymphocytic leukemia (CLL) [1–6].

Here we report a rare case of acute myeloid leukemia showing two clones, one clone being acute monoblastic leukemia (AMoL) with multiple trisomies and the other eventually becoming acute myeloid leukemia with myelodysplasia-related changes (AML-MRC) with monosomy 7.

## 2. Case Report

**Figure 1:**
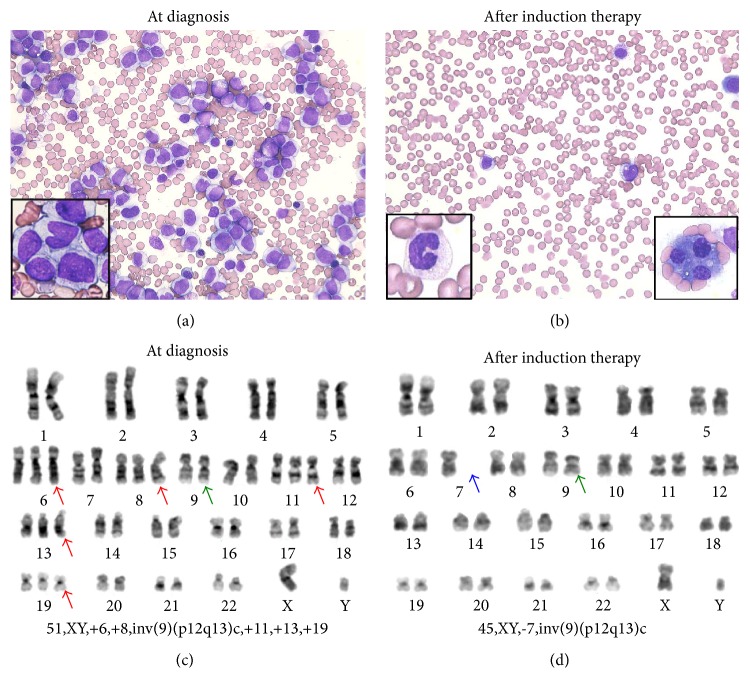
(a) and (b) May-Giemsa staining of a bone marrow smear before and after induction therapy. (a) Monoblasts (left lower panel) consisted of 82.4% of bone marrow mononuclear cells at diagnosis. (b) Blast population significantly decreased after induction therapy, but there were background differentiated cells showing a dysplastic feature including hypogranular neutrophils (left lower panel) and discrete multinuclear megakaryocytes (right lower panel). (c) and (d) G-banding of bone marrow before and after induction therapy. Each panel showed a dominant karyotype at each point. (c) In the initial sample, all cells were abnormal, with a dominant clone showing multiple trisomies (red arrows). (d) The clone with multiple trisomies completely disappeared, but the monosomy 7 (blue arrow) clone was unmasked after induction therapy. All cells showed a pericentric inversion of chromosome 9 (green arrow), a known normal variant in the general population.

The patient was a 45-year-old male. He had no prior history of chemotherapy, radiation therapy, or exposure to toxic substances. He was found to have anemia (Hb 8.9 g/dL) with a normal white blood cell (WBC) count (3.9 × 10^9^/L) at a periodic health check 2 months before admission. He visited a local clinic with the complaint of stomach ache 3 days before admission. He was then referred to our hospital due to fever and a high WBC count (73.7 × 10^9^/L). Peripheral blood count values were as follows: hemoglobin: 8.7 g/L (normal range: 13.4 to 17.6 g/L); WBC: 203.5 × 10^9^/L (normal range: 3.5 × 10^9^ to 9.3 × 10^9^/L) with 62.5% monoblast cells; platelets: 98 × 10^9^/L (normal range: 120 × 10^9^ to 400 × 10^9^/L); and serum lactate dehydrogenase level: 1675 U/L (normal range: 119 to 229 U/L). Bone marrow examination showed severe hypercellular marrow with 82% monoblasts that were positive for peroxidase staining and nonspecific esterase staining (Figure 1(a)). A diagnosis of acute monoblastic leukemia (AMoL) was made according to the WHO classification [7]. Flow cytometry revealed a population of monoblast cells that was positive for CD33, CD11b, CD14, CD15, and HLA-DR and was negative for CD13. Cytogenetic evaluation showed 51,XY,+6,+8,inv(9)(p12q13)c,+11,+13,+19[12]/52,idem,+Y[8] (Figure 1(c)). All of the 20 analysed cells in cytogenetics consisted of two related clones with multiple trisomies (Figure 1(c)). Interphase fluorescence in situ hybridization (FISH) analysis using chromosome 8 centromeric probes revealed 65% trisomy 8 cells in bone marrow (Table 1). FISH using MLL probes showed 3 signals reflecting chromosome 11 trisomy without break-apart signals.

The patient received induction therapy with idarubicin and cytarabine. Thrombocytopenia was sustained even after neutrophil recovery, and bone marrow aspiration on day 22 after induction therapy revealed 4.4% monoblasts with dysplastic differentiated cells including hypogranular neutrophils and micromegakaryocytes (Figure 1(b)). Karyotype after induction therapy was 45,XY,-7,inv(9)(p12q13)c[19]/46,XY,inv(9)(p12q13)c[1] (Figure 1(d)). FISH analysis using chromosome 7 and 8 centromeric probes revealed 73.4% monosomy 7 cells and 0.6% trisomy 8 cells.

To determine whether monosomy 7 clones existed in the initial sample as the primary clone or developed after induction therapy, FISH analysis was performed on a Carnoy-fixed bone marrow specimen that had been stored before induction therapy. Monosomy 7 cells accounted for 23.4% of the cells in the initial bone marrow specimen (Table 1). We concluded that two cytogenetically unrelated clones existed at diagnosis. The clone with multiple trisomies responded to induction therapy and disappeared, while the other clone with monosomy 7 persisted despite induction therapy. Despite a lower (4.4%) blast count after induction therapy, the monosomy 7 clone detected by FISH was disproportionally high (73.4%) with dysplastic cells. This observation means that the cells with monosomy 7 retained their differentiation ability and manifest, at least initially, as myelodysplasia. From these observations, we concluded that the cells with multiple trisomies represented de novo AMoL and the cells with monosomy 7 eventually became AML-MRC.

The patient underwent reinduction therapy with MEC (mitoxantrone, etoposide, and cytarabine), but there was no recovery to normal hematopoiesis. We considered that the majority of the remaining clone was MDS clone with monosomy 7 and we administered Azacitidine (AZA) as a third-line therapy. Although neutrophils slightly recovered, FISH analysis of bone marrow aspiration showed 84.4% monosomy 7 cells after AZA therapy (Table 1). He underwent related peripheral blood stem cell transplantation from his male cousin. After transplantation, both monosomy 7 and trisomy 8 were eliminated and the karyotype showed normal male 46,XY[20] without inv(9)(p12q13)c. On day 145 after transplantation, blast cells were detected in the peripheral blood, and bone marrow aspiration showed 45.4% blasts with 56.4% monosomy 7 cells without increase of trisomy 8 cells by FISH. Flow cytometry showed that the blast cells were positive for CD33, CD11b, CD11c, CD13, and HLA-DR and negative for CD14 and CD15. We diagnosed that only AML-MRC clone with monosomy 7 was relapsed after transplantation. Due to severe fungal infection, no further therapy was given for the relapsed leukemia, and he died 159 days after transplantation.

### 2.1. Banding Cytogenetics

At the time of diagnosis and during follow-up, standard cytogenetic G-banding analysis was performed on bone marrow cells, and the karyotype was described according to the latest version of International System for Human Cytogenetic Nomenclature [8].

### 2.2. FISH

Molecular cytogenetic studies of fluorescence in situ hybridization (FISH) were performed on interphase cells using centromeric probes for chromosomes 7, 8 and MLL (Vysis CEP7, CEP8 probe, and MLL Dual Color, Break-Apart Rearrangement probe, Abbott Molecular, Abbott Park, IL).

## 3. Discussion

Cytogenetic analysis is essential for establishing a diagnosis and for evaluation of treatment response in hematological malignancies. Clonal cytogenetic abnormalities were identified in about 50% of MDS cases [9] and 50–60% of adult AML cases [10]. In AML, cytogenetic subclones were detected in 15.8% patients. And the presence of cytogenetic subclones was known to be an adverse prognosis factor [11, 12]. In most cases, cytogenetic analyses show related clonal chromosome abnormalities in all abnormal cells. In exceptional cases, however, more than one clone has been identified in an individual case. The frequencies of cytogenetically independent clones found in an individual case are 4.3–6.5% in MDS and 1.1–3.7% in AML [1, 11]. In most reported cases with cytogenetically unrelated clones in hematological malignancy, two clones were detected in one sample simultaneously by G-band analysis either in the initial disease [1–3] or relapsed disease [3] or in secondary MDS or AML [4]. Raimondi et al. reported the appearance of an unrelated clone as relapsed leukemia 6–35 months after remission of the initial disease [5]. Our case is unique because the result of cytogenetic analyses was completely different before and after induction therapy. At the time of diagnosis, two “related” clones with multiple trisomies were found in all of the 20 analysed cells in G-banding (Figure 1(b)). Simultaneous existence of two clones was only confirmed retrospectively by FISH analysis. Because cells need to be induced into metaphase when analysed by the G-banding method, G-banding preferentially detects cells in rapid cell cycles such as an acute leukemic clone. It is not surprising that the less mitotically active MDS clone represented by monosomy 7 was overgrown by the rapidly dividing leukemic clone with multiple trisomies in the conventional cytogenetic study. In contrast, interphase FISH detected both populations of cells and allowed for easy quantification of the relative numbers of cells in each clone [13].

Furthermore, most of the previously reported cases had two unrelated clones showing the same hematological disease. There was one reported case of initially diagnosed MDS RA with trisomy 8 in a 61-year-old female who developed AML-M2 with trisomy 21 without trisomy 8 at 6 months after the initial diagnosis [6]. In that case, coexistence of two independent clones was not verified by FISH in initial sample. In our case, simultaneously developed two clones showed different disease status and different responses to chemotherapy. Two months before admission, he was diagnosed with anemia but with a normal WBC at a periodical health check. This suggests that the monosomy 7 clone, which has MDS-like features, existed, while the multiple trisomies clone, which has AML-like features, was still not predominant. As for the response to chemotherapy, the multiple trisomies clone responded to induction therapy and was eliminated, while the monosomy 7 clone resisted chemotherapy and persisted as MDS. Hematological stem cell transplantation transiently eliminated both clones, but the monosomy 7 clone relapsed after transplantation as blastic phase of AML-MRC.

Cytogenetically unrelated clones might originate from different diseases that developed coincidentally at the same time, but they can also be derived from a common ancestral premalignant cell. Stark et al. reported a case of AML in which spectral karyotyping (SKY) FISH analysis revealed two apparently unrelated clones that had common underlying aberration [14]. In our case, it was possible that the two clones shared cryptic subbanding cytogenetic or molecular aberration, though we could not verify it.

Pericentric inversion of chromosome 9 is a known normal constitutional variant (polymorphism) seen in normal humans. Its frequency is estimated to be 0.8 to 2% in the general population and it is inherited in a Mendelian fashion without any clinical significance [15, 16]. Although some reports suggested that inv(9) was involved in infertility and recurrent abortion [17–19], no functional effect on hematological malignancies was reported [20].

Here we report a rare case of acute myeloid leukemia with two cytogenetically unrelated clones, in which one clone was de novo AMoL with multiple trisomies and the other was AML-MRC with monosomy 7. Longitudinal assessment of clonal percentage of each clone by FISH analysis was useful for evaluating the disease status and the response to therapy.

## Figures and Tables

**Table 1:** Summary of bone marrow, FISH, and cytogenetic findings.

Timing	Blast (%)	FISH		Karyotype
		^*∗*^CEP ×3 (normal: 0.0–2.0%)	^*∗∗*^CEP7 ×1 (normal: 0.0–6.0%)	
At diagnosis	82.4	65% [325/500]	23.4% [117/500]	51,XY,+6,+8,inv(9)(p12q13)c,+11,+13,+19[12]/52,idem,+Y[8]

After induction (IDA-AraC)	3.2	0.6% [3/500]	73.4% [367/500]	45,XY,-7,inv(9)(p12q13)c[19]/46,XY,inv(9)(p12q13)c[1]

After consolidation (Mit-VP16-AraC)	5.4	1.2% [6/500]	65.8% [329/500]	45,XY,-7,inv(9)(p12q13)c[18]/46,XY,inv(9)(p12q13)c[2]

After Azacytidine	4.4	0.6% [3/500]	84.4% [422/500]	n/a

After HSCT	0.2	0.8% [4/500]	0.6% [3/500]	46,XY[20]

Relapse	45.4	0.2% [1/500]	56.4% [282/500]	n/a

IDA: Idamycin; AraC: cytarabine; Mit: mitoxantrone; VP16: etoposide; HSCT: Hematopoietic Stem Cell Transplantation.

^*∗*^Centromere probe for chromosome 7. ^*∗∗*^Centromere probe for chromosome 8.
